# Metabolite Profile and Biological Activities of *Zanthoxylum nemorale*: A Metabolomic and Multivariate Statistical Analysis Study

**DOI:** 10.1002/cbdv.71883

**Published:** 2026-10-27

**Authors:** Victor S. A. de Lira, Floricéa M. Araújo, Rodrigo N. Santos, Lourdes C. Souza‐Neta, Leonardo Zambotti‐Villela, Pio Colepicolo, Lidyanne Y. S. Aona, Paulo R. Ribeiro

**Affiliations:** ^1^ Metabolomics Research Group, Instituto de Química Universidade Federal da Bahia Salvador Brazil; ^2^ Departamento de Ciências Exatas e da Terra I Universidade do Estado da Bahia Salvador Brazil; ^3^ Departamento de Bioquímica, Instituto de Química Universidade de São Paulo São Paulo Brazil; ^4^ Federal University of Recôncavo da Bahia Cruz das Almas Bahia Brazil

**Keywords:** antioxidant and antimicrobial activities, bioactive metabolites, metabolite profiling, Rutaceae

## Abstract

This study investigated the metabolite profile and biological activities of *Zanthoxylum nemorale* leaf and stem extracts obtained using hexane, ethyl acetate, and methanol by combining untargeted LC‐HRMS metabolomics, multivariate statistical analysis, antioxidant, and antimicrobial assays. A total of 112 metabolites were putatively identified, including coumarins, lignans, flavonoids, alkaloids, phenolic acids, terpenoids, and fatty acids. Methanol extracts exhibited the strongest antioxidant activity, with IC_50_ values of 53.12 ± 1.11 and 60.70 ± 0.28 µg mL^−^^1^ for stem and leaf extracts, respectively, and showed the highest antibacterial activity against *Staphylococcus aureus* and *Bacillus cereus*. Chemometric analyses (PCA, PLS‐DA, and hierarchical clustering) clearly discriminated the extracts according to plant organ and solvent polarity. Overall, the extracts were more effective against Gram‐positive bacteria than Gram‐negative bacteria and fungi. These findings highlight *Z. nemorale* as a promising source of antioxidant and antimicrobial phytochemicals and demonstrate the value of metabolomics for linking chemical composition with biological activity.

## Graphical abstract

An integrated LC‐HRMS metabolomics and bioactivity approach reveals the chemical diversity and biological potential of *Zanthoxylum nemorale*. Extraction solvent and plant organ markedly influence metabolite profiles and bioactivity, with methanolic extracts exhibiting pronounced antioxidant and antibacterial activities, particularly against Gram‐positive bacteria. These findings highlight *Z. nemorale* as a promising source of bioactive natural products.

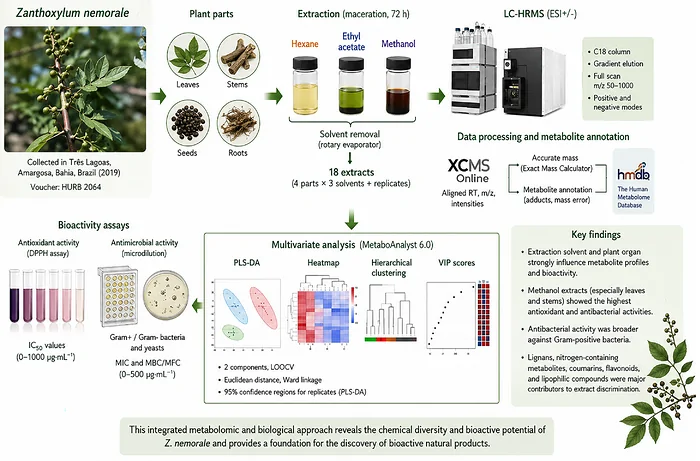

**Abbreviations**
ZNLH: *Z. nemorale* leaf extract in hexane
ZNSTH: *Z. nemorale* stem extract in hexane
ZNLEA: *Z. nemorale* leaf extract in ethyl acetate
ZNSTEA: *Z. nemorale* stem extract in ethyl acetate
ZNLM: *Z. nemorale* leaf extract in methanol
ZNSTM: *Z. nemorale* stem extract in methanol
MBC: Minimal bactericidal concentration
MIC: Minimal inhibitory concentration
na: not active
nt: not tested

## 1 Introduction

Natural products remain one of the most important sources of biologically active compounds for the discovery of new therapeutic agents. In this context, plants represent an invaluable reservoir of structurally diverse secondary metabolites that directly or indirectly shape the currently available pharmaceuticals [1]. Plant‐derived secondary metabolites, including alkaloids, flavonoids, phenolic acids, terpenoids, coumarins, lignans, and amides, have attracted considerable attention because of their pharmacological properties, which include antioxidant, antimicrobial, anti‐inflammatory, and anticancer potential. Furthermore, the growing concern regarding the development of antimicrobial resistance, together with increasing consumer demand for naturally derived antioxidants and bioactive ingredients, has further stimulated interest in the systematic investigation of pharmacological potential of medicinal plants [2].

The genus *Zanthoxylum* (Rutaceae) comprises species distributed across tropical and subtropical regions, several of which have recognized ethnobotanical importance and are traditionally used for medicinal purposes. *Z. nemorale* occurs in Brazil and represents a potentially valuable source of chemically diverse secondary metabolites. Nevertheless, information concerning its specific ethnobotanical applications, ecological characteristics, and phytochemical composition remains limited. In this context, investigating its metabolomic profile and biological activities may contribute to a better understanding of the chemical diversity of this comparatively understudied species. Several species have long been used in traditional medicine for the treatment of infections, inflammatory disorders, gastrointestinal ailments, pain, fever, and parasitic diseases, which reflects the broad medicinal interest in this genus [3]. Phytochemical investigations have shown that *Zanthoxylum* species are important sources of benzophenanthridine alkaloids, quinoline alkaloids, lignans, coumarins, flavonoids, amides, terpenoids, and essential oils, which are associated with a broad spectrum of biological activities, including antimicrobial, antioxidant, cytotoxic, anti‐inflammatory, insecticidal, and antiparasitic effects [4]. For example, one benzophenanthridine alkaloid and five known alkaloids were isolated from the stem bark of *Z. rhetsa* and showed notable cytotoxic and antimicrobial activities [5], whereas a new acyclic monoterpene and eleven known alkaloids isolated from the pericarps of *Z. bungeanum* showed strong antibacterial activity [6]. However, despite the extensive phytochemical and pharmacological studies conducted on several *Zanthoxylum* species, relatively little information is available regarding *Zanthoxylum nemorale [* 7 *]*. Consequently, its metabolite composition remains poorly characterized, and the relationship between its chemical constituents and biological activities has not been comprehensively investigated yet.

Recent advances in metabolomics have enabled the comprehensive characterization of complex plant extract metabolomes using high‐resolution analytical platforms such as liquid chromatography coupled with high‐resolution mass spectrometry (LC‐HRMS). When combined with multivariate statistical analyses, metabolomics approaches allow the discrimination of samples according to species, plant organ, geographical origin, or extraction method while simultaneously identifying metabolites associated with biological activities. Thus, becoming a powerful tool for biomarker discovery and for establishing chemical–biological relationships in medicinal plants [8, 9]. To the best of our knowledge, this is the first study to integrate LC‐HRMS‐based metabolomic profiling, multivariate statistical analysis, and antioxidant and antimicrobial assays to investigate the chemical diversity and biological activities of leaf and stem extracts of *Z. nemorale*. To investigate the antibacterial potential of *Z. nemorale*, representative Gram‐positive and Gram‐negative bacteria were selected, including *Staphylococcus aureus*, *Bacillus subtilis*, *B. cereus*, *Micrococcus luteus*, *Escherichia coli*, and *Pseudomonas aeruginosa*. These microorganisms represent distinct bacterial groups with different cell envelope characteristics and susceptibility profiles. The inclusion of Gram‐positive and Gram‐negative bacteria enables a preliminary evaluation of the antibacterial spectrum of the extracts, while the selection of clinically relevant and commonly used reference strains facilitates comparison with previous studies on plant‐derived antimicrobial agents. Furthermore, a multivariate statistical approach was used to further investigate the relationship between the chemical composition and biological activities of the extracts.

## 2 Results

This study provides an integrated characterization of the metabolite profile and biological activities of *Z. nemorale*, combining untargeted metabolomics, multivariate analysis, and antioxidant and antimicrobial assays. The results reveal marked chemical and biological differentiation according to extraction solvent and plant organ and identify metabolite classes potentially associated with the observed bioactivities.

*Z. nemorale* extracts exhibited markedly different antioxidant activities, with IC_50_ values ranging from 53.12 ± 1.11 to 944.24 ± 44.43 µg/mL (Table 1). The stem methanol extract (ZNSTM) showed the strongest activity (IC_50_ = 53.12 ± 1.11 µg/mL), closely followed by the leaf methanol extract (ZNLM; 60.70 ± 0.28 µg/mL) and the leaf ethyl acetate extract (ZNLEA; 69.53 ± 1.81 µg/mL). In contrast, the leaf hexane extract (ZNLH) showed moderate activity (119.11 ± 11.68 µg/mL), whereas the stem ethyl acetate extract (ZNSTEA) exhibited the weakest activity (944.24 ± 44.43 µg/mL), and no detectable activity was observed for the stem hexane extract (ZNSTH). Gallic acid, used as the reference antioxidant, showed an IC_50_ of 1.21 ± 0.06 µg/mL (Table 1).

**TABLE 1:** Antioxidant activity of *Z. nemorale* extracts determined by the DPPH radical‐scavenging assay.

Extracts	DPPH radical‐scavenging activity (IC_50_, µg mL^−^^1^)
**ZNLH**	119.11 ± 11.68
**ZNSTH**	>1000
**ZNLEA**	69.53 ± 1.81
**ZNSTEA**	944.24 ± 44.43
**ZNLM**	60.70 ± 0.28
**ZNSTM**	53.12 ± 1.11
**gallic acid**	1.21 ± 0.06

Values are expressed as mean ± standard deviation (SD) of triplicate measurements. Gallic acid was used as the positive control. IC_50_ represents the concentration required to reduce the DPPH radical concentration by 50%.

The antioxidant activity of *Z. nemorale* was strongly influenced by solvent polarity and, to a lesser extent, by plant organ. The comparable activities of the stem and leaf methanol extracts suggest that antioxidant constituents are present in both tissues and are preferentially recovered in polar fractions. These metabolites may include phenolic and other hydroxyl‐containing compounds capable of stabilizing radicals through hydrogen‐atom or electron transfer. Nevertheless, the strong activity of the leaf ethyl acetate extract (ZNLEA) indicates that relevant antioxidant constituents are also present among moderately polar metabolites.

The influence of plant organ was particularly evident in the ethyl acetate fractions, as ZNLEA exhibited an IC_50_ nearly 14‐fold lower than the corresponding stem extract (69.53 vs. 944.24 µg/mL). This pronounced difference suggests an organ‐dependent distribution and/or composition of moderately polar antioxidant metabolites. In contrast, the moderate activity of the leaf hexane extract and the absence of detectable activity in the stem hexane extract indicate a comparatively limited contribution of nonpolar constituents to the radical‐scavenging capacity of *Z. nemorale*. Although the most active extract, ZNSTM, was approximately 44‐fold less potent than gallic acid, this difference should be interpreted considering that crude extracts contain complex mixtures of compounds, whereas gallic acid is a purified antioxidant. Overall, the predominance of antioxidant activity in the methanol extracts, together with the strong activity of ZNLEA, indicates that the radical‐scavenging potential of *Z. nemorale* is mainly associated with polar to moderately polar constituents.

The antimicrobial activity of *Z. nemorale* also varied according to plant organ and extraction solvent, with a clear predominance of activity against Gram‐positive bacteria (Tables 2 and 3). Among the tested microorganisms, *S. aureus* and *B. cereus* were the most susceptible. ZNSTM showed particularly relevant bactericidal activity against *S. aureus* (MIC = MBC = 62.5 µg/mL), whereas ZNLM was bactericidal against *B. cereus* at the same concentration. ZNSTM also inhibited B. subtilis and *B. cereus* (MIC = 125 µg/mL), highlighting the antimicrobial potential of the methanol fractions.

**TABLE 2:** Antibacterial activity of *Z. nemorale* extract.

Extracts	Gram‐positive bacteria							
	*S. aureus*		*M. luteus*		*B. subtilis*		*B. cereus*	
	MIC	MBC	MIC	MBC	MIC	MBC	MIC	MBC
**ZNLH**	62.5	—	—	—	—	—	125	500
**ZNSTH**	125	—	—	—	125	—	—	—
**ZNLEA**	—		500	—	—	—	500	—
**ZNSTEA**	125	250	500	—	—	—	—	—
**ZNLM**	—	—	—	—	—	—	62.5	62.5
**ZNSTM**	62.5	62.5	—	—	125	—	125	—
**benzylpenicillin**	0.19	0.19	0.78	0.78	0.08	0.08	25	25

Minimal Bactericidal Concentration (MBC) and Minimal Inhibitory Concentration (MIC) Are Expressed in µg.mL^−1.^

**TABLE 3:** Results of antimicrobial activity against gram‐negative bacteria and fungi for *Z. nemorale* extract.

Extracts	Gram‐negative bacteria		Fungi	
	*E. coli*	*P. aeruginosa*	*C. albicans*	*C. glabrata*
	MIC	MIC	MIC	MIC
**ZNLH**	—	—	—	—
**ZNSTH**	—	—	—	—
**ZNLEA**	500	500	—	500
**ZNSTEA**	—	—	—	—
**ZNLM**	—	—	—	—
**ZNSTM**	—	—	250	—
**gentamicin**	3.1	1.25	—	—
**ciclopirox**	—	—	12.5	12.5

Nonpolar and moderately polar extracts also contributed to the antibacterial profile. ZNLH inhibited *S. aureus* (MIC = 62.5 µg/mL) and *B. cereus* (MIC = 125 µg/mL), although its bactericidal activity was considerably weaker or absent, indicating that antimicrobial constituents are not restricted to polar fractions. Ethyl acetate extracts showed a distinct profile: ZNSTEA was bactericidal against *S. aureus* (MIC = 125 µg/mL; MBC = 250 µg/mL), while ZNLEA displayed the broadest spectrum of activity, including weak inhibition of the Gram‐negative bacteria *E. coli* and *P. aeruginosa* (MIC = 500 µg/mL). The substantially lower susceptibility of Gram‐negative bacteria compared with Gram‐positive species may be partly related to their outer membrane, which restricts the penetration of many plant‐derived compounds. Antifungal activity was limited, with inhibition observed only for ZNSTM against *C. albicans* and ZNLEA against *C. glabrata* (Table 3). As expected, the reference antimicrobial agents were considerably more potent than the crude extracts.

Interestingly, the methanol extracts displaying the strongest bactericidal effects were also among the most active in the antioxidant assay. ZNSTM exhibited the highest antioxidant activity and was bactericidal against S. aureus, whereas ZNLM showed similarly strong antioxidant activity and bactericidal activity against *B. cereus*. Although these parallel responses do not establish that the same compounds are responsible for both effects, they suggest that polar metabolites recovered in the methanol fractions may contribute to both biological activities. Conversely, the antibacterial activity of ZNLH despite its lower antioxidant capacity indicates that the chemical determinants of antioxidant and antimicrobial activities are not necessarily identical.

Untargeted LC‐HRMS analysis enabled the detection and tentative annotation of 112 metabolomic features in *Z. nemorale* extracts based on accurate mass measurements, molecular formulas, adduct information, isotopic patterns, and database comparisons, with mass errors generally below 10 ppm (Table 4). These annotations provide an overview of the chemical diversity of the extracts, including coumarins, lignans, flavonoids, alkaloids, phenolic acids, terpenoids, and fatty acids. Since MS/MS fragmentation data and authentic standards were not available, the proposed structures should be regarded as tentative. Where applicable, possible isomeric structures were considered, and the annotations should not be interpreted as unequivocal structural identifications (Table 4 and Figures 1–4). The chemical profile revealed considerable metabolite diversity, comprising several classes of specialized metabolites. Coumarins and related compounds were prominently represented, including umbelliferone (**1**), scopoletin (**3**), auraptene (**5**), bergapten (**11**), xanthyletin (**17**), and several prenylated and methoxylated derivatives. Flavonoids and related phenolics, such as kaempferol (**24**), isoquercitrin (**26**), apigenin (**27**), diosmetin (**28**), and diosmin (**29**), were also detected, together with phenolic acids, including p‐hydroxybenzoic (**49**), protocatechuic (**50**), gallic (**52**), and caffeic (**55**) acids. Lignans constituted another important group, represented by syringaresinol (**35**), pinoresinol (**36**), sesamin (**39**), fargesin (**40**), and related structures. In addition, several nitrogen‐containing metabolites, including furoquinoline, benzophenanthridine, and other putative alkaloids, as well as fatty acids, terpenoid‐related metabolites, and other lipophilic constituents, were annotated.

**TABLE 4:** Putatively annotated metabolites in *Z. nemorale* extracts based on LC‐HRMS analysis. Names, molecular formulas, adducts, and high‐resolution mass (*m/z*) used for identification are given.

Number	Metabolite name	Formula	Adduct	High‐resolution mass (*m/z*) Expected	High‐resolution mass (*m/z*) experimental	Error (ppm)
1	Umbelliferone + 1 ISO*	C_9_H_6_O_3_	[M+H]^+^	163.0395	163.039	3.07
2	Lacinartin	C_15_H_16_O_4_	[M+H]^+^	261.1127	261.113	1.15
3	Scopoletin + 1 ISO*	C_10_H_8_O_4_	[M+H]^+^	193.0501	193.0499	1.04
4	7‐metoxicumarina	C_10_H_8_O_3_	[M+H]^+^	177.0551	177.0551	0
5	auraptene	C_19_H_22_O_3_	[M+H]^+^	299.1647	299.1621	8.69
6	6,7,8‐trimethoxycoumarin + 2 ISO*	C_12_H_12_O_5_	[M‐H]^−^	235.0607	235.0608	0.43
7	7,8‐ dimethoxycoumarin + 1 ISO*	C_11_H_10_O_4_	[M‐H]^−^	205.0501	205.0497	1.95
8	6,8‐dimethoxy‐7‐O‐prenylcoumarin + 1 ISO*	C_16_H_18_O_5_	[M‐H]^−^	289.1076	289.1049	9.34
9	Phytodolor	C_11_H_10_O_5_	[M‐H]^−^	221.045	221.0452	0.9
10	8‐methoxy auraptene + 2 ISO*	C_20_H_24_O_4_	[M+Na]^+^	351.1572	351.157	0.57
11	Bergapten	C_12_H_8_O_4_	[M+Na]^+^	239.032	239.0319	0.42
12	7‐[(E)‐3′,7′‐dimethyl‐6′‐oxo‐2′,7′‐octadienyl]oxycoumarin	C_19_H_20_O_4_	[M+H]^+^	313.144	313.1438	0.64
13	7‐((2′E,5′E)‐7′‐Hydroxy‐3′,7′‐dimethylocta‐2′,5′‐dienyloxy)coumarin + 1 ISO*	C_19_H_22_O_4_	[M+H]^+^	315.1596	315.1578	5.71
14	Schinilenol + 1 ISO*	C_20_H_24_O_5_	[M+Na]^+^	367.1522	367.1523	0.27
15	Methylschinilenol + 2 ISO*	C_21_H_26_O_5_	[M‐H]^−^	357.1702	357.1702	0
16	Schinindiol	C_20_H_26_O_6_	[M+Na]^+^	385.1627	385.1621	1.56
17	Xanthyletin	C_14_H_12_O_3_	[M+H]^+^	229.0865	229.0863	0.87
18	Xanthoxyletin + 2 ISO*	C_15_H_14_O_4_	[M+H]^+^	259.097	259.0944	10.03
19	Avicennol	C_20_H_22_O_5_	[M+Na]^+^	365.1365	365.1373	2.19
20	Avicennol methyl ether + 1 ISO*	C_21_H_24_O_5_	[M+Na]^+^	379.1522	379.1525	0.79
21	Marmesin + 1 ISO*	C_14_H_14_O_4_	[M‐H]^−^	245.0814	245.0813	0.41
22	Felopterina	C_17_H_16_O_5_	[M‐H]^−^	299.092	299.0892	9.36
23	4′‐O‐Methylkanzonol W	C_21_H_18_O_5_	[M+H]^+^	351.1233	351.1226	1.99
24	Kaempferol	C_15_H_10_O_6_	[M‐H]^−^	285.0399	285.04	0.35
25	Afzelin + 2 ISO*	C_21_H_20_O_10_	[M‐H]^−^	431.0978	431.0989	2.55
26	Isoquercitrin + ISO*	C_21_H_20_O_12_	[M‐H]^−^	463.0877	463.0882	1.08
27	Apigenin	C_15_H_10_O_5_	[M‐H]^−^	269.045	269.0451	0.37
28	Diosmetin	C_16_H_12_O_6_	[M‐H]^−^	299.0556	299.0544	4.01
29	Diosmin	C_28_H_32_O_15_	[M‐H]^−^	607.1663	607.1665	0.33
30	Homo‐esperetin‐7‐O‐rutinoside	C_28_H_34_O_15_	[M‐H]^−^	609.182	609.1821	0.16
31	Cyanidin 3‐(6″‐acetylglucoside)	C_23_H_23_O_12_	[M‐H]^−^	490.1111	490.1112	0.2
32	Lichexanthone	C_16_H_14_O_5_	[M‐H]^−^	285.0763	285.0762	0.35
33	5′,5″‐didemethoxypinoresinol	C_19_H_20_O_3_	[M+Na]^+^	319.131	319.1315	1.57
34	Eudesmin	C_24_H_30_O_6_	[M+H]^+^	415.2121	415.2126	1.2
35	Syringaresinol	C_22_H_26_O_8_	[M‐H]^−^	417.155	417.155	0
36	Pinoresinol	C_20_H_22_O_6_	[M‐H]^−^	357.1338	357.1338	0
37	(+)‐lariciresinol	C_20_H_24_O_6_	[M+H]^+^	361.1651	361.1631	5.54
38	Lignan	C_25_H_30_O_8_	[M‐H]^−^	457.1863	457.1872	1.97
39	Sesamin + 3 ISO*	C_20_H_18_O_6_	[M+H]^+^	355.1182	355.1178	1.13
40	Fargesin + 3 ISO*	C_21_H_22_O_6_	[M+Na]^+^	393.1314	393.1315	0.25
41	Dimethoxysamin	C_14_H_18_O_5_	[M+H]^+^	267.1232	267.1226	2.25
42	Zanthpodocarpin A	C_41_H_42_O_14_	[M‐H]^−^	757.2496	757.253	4.49
43	Citrusin B	C_27_H_36_O_13_	[M‐H]^−^	567.2078	567.2123	7.93
44	Glaberide I	C_14_H_16_O_6_	[M‐H]^−^	279.0869	279.0894	8.96
45	Utilines B	C_21_H_26_O_8_	[M‐H]^−^	405.155	405.1572	5.43
46	Isopentenyl ether of pluviatol	C_25_H_28_O_6_	[M+Na]^+^	447.1784	447.178	0.89
47	(E)‐4‐(4‐hydroxy‐3‐methylbut‐2‐enyl‐oxy)benzaldehyde + 1 ISO*	C_12_H_14_O_3_	[M+Na]^+^	229.0841	229.0863	9.6
48	(E)‐4‐(4‐hydroxy‐3‐methylbut‐2‐enyl‐oxy)benzoic acid + 1 ISO*	C_12_H_14_O_4_	[M+Na]^+^	245.079	245.0811	8.57
49	p‐hydroxy‐benzoic acid	C_7_H_6_O_3_	[M‐H]^−^	137.0239	137.0242	2.19
50	Protocatechuic acid	C_7_H_6_O_4_	[M‐H]^−^	153.0187	153.0184	1.96
51	Dioxamin + 1 ISO*	C_10_H_12_O_4_	[M+H]^+^	197.0814	197.081	2.03
52	Gallic acid	C_7_H_6_O_5_	[M‐H]^−^	169.0137	169.0134	1.78
53	Syringaldehyde	C_9_H_10_O_4_	[M‐H]^−^	181.0501	181.0487	7.73
54	Dodecyl caffeate	C_21_H_32_O_4_	[M‐H]^−^	347.2222	347.2201	6.05
55	Caffeic acid	C_9_H_8_O_4_	[M‐H]^−^	179.0344	179.0342	1.12
56	Sinapaldehyde	C_11_H_12_O_4_	[M‐H]^−^	207.0657	207.0655	0.97
57	N1‐trans‐Feruloylagmatine	C_15_H_22_N_4_O_3_	[M+Na]^+^	329.159	329.1582	2.43
58	Wutaipyranol A + 1 ISO*	C_14_H_16_O_3_	[M+Na]^+^	255.0997	255.1022	9.8
59	Irenolone + 1 ISO*	C_19_H_12_O_3_	[M+H]^+^	289.0865	289.0859	2.08
60	2‐Hydroxy‐4‐(4‐methoxyphenyl)‐1H‐phenalen‐1‐one	C_20_H_14_O_3_	[M+H]^+^	303.1021	303.1017	1.32
61	Linanyl acetate	C_12_H_20_O_2_	[M‐H]^−^	195.1385	195.1382	1.54
62	Tagetonol + 1 ISO*	C_10_H_18_O_2_	[M‐H]^−^	169.1229	169.1224	2.96
63	Monomenthyl succinate	C_14_H_24_O_4_	[M‐H]^−^	255.1596	255.1603	2.74
64	Sesquichamaenol	C_15_H_22_O_2_	[M+Na]^+^	257.1518	257.1519	0.39
65	Dihydroartemisinin + 1 ISO*	C_15_H_24_O_5_	[M‐H]^−^	283.1546	283.1551	1.77
66	Macrocarpal I	C_28_H_42_O_7_	[M‐H]^−^	489.2852	489.2862	2.04
67	N‐Methylrosmaricine	C_21_H_29_NO_4_	[M‐H]^−^	358.2018	358.2022	1.12
68	Phytal	C_20_H_38_O	[M+NH_4_]^+^	312.3266	312.3259	2.24
69	gibberellin monocarboxylic acid + 5 ISO*	C_19_H_22_O_5_	[M+Na]^+^	353.1365	353.1385	5.66
70	(13R,14R)‐7‐Labdene‐13,14,15‐triol	C_20_H_36_O_3_	[M‐H]^−^	323.2586	323.2589	0.93
71	Avenestergenin B2	C_37_H_52_O_6_	[M‐H]^−^	591.3686	591.3692	1.01
72	7a,12a‐Dihydroxy‐3‐oxo‐4‐cholenoic acid	C_24_H_36_O_5_	[M‐H]^−^	403.2485	403.2491	1.49
73	6‐Deoxohomodolichosterone	C_29_H_50_O_4_	[M‐H]^−^	461.3631	461.3633	0.43
74	Choleston‐3‐ol, 2‐methylene‐[3β,5α] + 1 ISO*	C_28_H_48_O	[M+H]^+^	401.3783	401.3804	5.23
75	Norchelerythrine + 1 ISO*	C_20_H_15_NO_4_	[M+H]^+^	334.1079	334.1071	2.39
76	8‐hydroxy‐4, 7‐dimetoxy‐furoquinoline + 1 ISO*	C_13_H_11_NO_4_	[M+H]^+^	246.0766	246.0767	0.41
77	Skimmianine + 1 ISO*	C_14_H_13_NO_4_	[M+H]^+^	260.0923	260.0921	0.77
78	3‐hydroxydehydroevodiamine	C_19_H_16_N_3_O_2_^+^	[M+H]^+^	319.1321	319.1315	1.88
79	Zanthoaustrone C	C_19_H_21_NO_4_	[M+H]^+^	328.1549	328.1555	1.83
80	Cuscohygrine	C_13_H_24_N_2_O	[M+H]^+^	225.1967	225.1963	1.78
81	Cocculine	C_17_H_21_NO_2_	[M‐H]^−^	270.1494	270.1497	1.11
82	codeinone	C_18_H_19_NO_3_	[M‐H]^−^	296.1287	296.1286	0.34
83	Glaucine + 1 ISO*	C_21_H_25_NO_4_	[M‐H]^−^	354.1705	354.1712	1.98
84	(S)‐Laudanosine	C_21_H_27_NO_4_	[M‐H]^−^	356.1862	356.1869	1.97
85	Thalifoline	C_11_H_13_NO_3_	[M‐H]^−^	206.0817	206.0816	0.49
86	Indole‐3‐carboxaldehyde	C_9_H_7_NO	[M‐H]^−^	144.0449	144.0446	2.08
87	Palmitic acid	C_16_H_32_O_2_	[M+Na]^+^	279.23	279.2313	4.66
88	Hexadec‐3‐enoic acid	C_16_H_30_O_2_	[M+Na]^+^	277.2143	277.2169	9.38
89	2,3,4‐trihydroxy‐octadec‐2‐enoic acid	C_18_H_34_O_5_	[M+H]^+^	331.2484	331.2463	6.34
90	12‐Hydroxydodecanoic acid + 3 ISO*	C_12_H_24_O_3_	[M+NH_4_]^+^	234.2069	234.2062	2.99
91	22‐Hydroxydocosanoic acid + 1 ISO*	C_22_H_44_O_3_	[M‐H]^−^	355.3212	355.3219	1.97
92	24‐Hydroxytetracosanoic acid	C_24_H_48_O_3_	[M+NH_4_]^+^	402.3947	402.3941	1.49
93	N‐isobutyl‐(2E,4E)‐2,4‐tetradecadienamide	C_18_H_33_NO	[M+H]^+^	280.264	280.2625	5.35
94	Methyl palmitate + ISO*	C_17_H_34_O_2_	[M‐H]^−^	269.2481	269.2483	0.74
95	Ethyl palmitate	C_18_H_36_O_2_	[M‐H]^−^	283.2637	283.264	1.06
96	Heptadecanal	C_17_H_34_O	[M+NH_4_]^+^	272.2953	272.2948	1.84
97	Tricosanal	C_23_H_46_O	[M‐H]^−^	337.347	337.3474	1.19
98	Dihydroergotamine + 1 ISO*	C_16_H_17_NO_2_	[M+H]^+^	256.1338	256.1334	1.56
99	Planispine A	C_20_H_16_O_5_	[M+H]^+^	337.1076	337.1084	2.37
100	8‐hydroxy‐9‐methoxy‐2,3‐(methylenedioxy) benzophenanthridine	C_20_H_14_O_4_	[M+H]^+^	319.097	319.0967	0.94
101	2‐Pentadecylfuran	C_19_H_34_O	[M+NH_4_]^+^	296.2953	296.2945	2.7
102	Musanolone E	C_19_H_12_O_4_	[M+H]^+^	305.0814	305.0808	1.97
103	Phytosphingosine	C_18_H_39_NO_3_	[M+Na]^+^	340.2828	340.2823	1.47
104	(S)‐[8]‐Gingerol	C_19_H_30_O_4_	[M+Na]^+^	345.2042	345.2032	2.9
105	Isopropyl apiosylglucoside	C_14_H_26_O_10_	[M+H]^+^	355.1604	355.1601	0.84
106	Piperundecalidine	C_23_H_29_NO_3_	[M+Na]^+^	390.2045	390.2038	1.79
107	(3S,5R,6R,7E)‐3,5,6‐Trihydroxy‐7‐megastigmen‐9‐one	C_13_H_22_O_4_	[M‐H]^−^	241.144	241.1444	1.66
108	[8]‐Paradol	C_19_H_30_O_3_	[M‐H]^−^	305.2117	305.212	0.98
109	[12]‐Shogaol	C_23_H_36_O_3_	[M‐H]^−^	359.2586	359.2593	1.95
110	Harpagoside	C_24_H_30_O_11_	[M‐H]^−^	493.171	493.1718	1.62
111	Quinic acid	C_7_H_12_O_6_	[M‐H]^−^	191.0556	191.0557	0.52
112	Nitidanin	C_21_H_24_O_8_	[M‐H]^−^	403.1393	403.1416	5.71

*possible isomers

All metabolite assignments are tentative and based on accurate mass, molecular formula, adduct information, and database comparisons. Possible isomers are indicated where applicable. Definitive structural confirmation requires additional analytical evidence, including MS/MS fragmentation and/or authentic standards.

PLS‐DA revealed distinct metabolomic profiles among the six *Z. nemorale* extracts (Figure 1a). Components 1 and 2 accounted for 43.8% and 16.8% of the variation represented by the model, respectively. Methanol extracts were separated from hexane extracts primarily along Component 1, whereas ethyl acetate extracts occupied intermediate positions. Differences between leaf and stem extracts were also observed, suggesting that both extraction solvent and plant organ influenced the metabolomic profiles. The PLS‐DA model was evaluated using LOOCV, yielding R^2^ and Q^2^ values of 0.93 and 0.85, respectively. These findings were interpreted in conjunction with hierarchical clustering and VIP analysis to investigate chemical differences among the extracts. Given the exploratory nature of the study, the discriminating features were considered candidate chemical markers rather than independently validated biomarkers.

**FIGURE 1:**
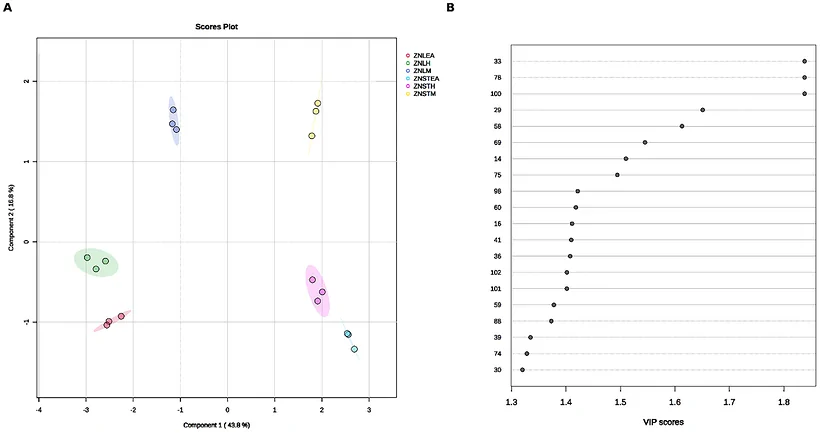
(a) Partial least squares discriminant analysis (PLS‐DA) and (b) Variable Importance in Projection (VIP) scores based on the compounds identified in *Z. nemorale* extracts. **33)** 5′,5″‐Didemethoxypinoresinol, **78)** 3‐Hydroxydehydroevodiamine, **100)** 8‐Hydroxy‐9‐methoxy‐2,3‐(methylenedioxy)benzophenanthridine, **29)** Diosmin, **58)** Wutaipyranol A, **69)** Gibberellin monocarboxylic acid, **14)** Schinilenol, **75)** Norchelerythrine, **98)** Dihydroergotamine, **60)** 2‐Hydroxy‐4‐(4‐methoxyphenyl)‐1H‐phenalen‐1‐one, **16)** Schinindiol, **41)** Dimethoxysamin, **36)** Pinoresinol, **102)** Musanolone E, **101)** 2‐Pentadecylfuran, **59)** Irenolone, **88)** Hexadec‐3‐enoic acid, **39)** Sesamin, **74)** Choleston‐3‐ol, 2‐methylene‐[3β,5α], **30)** Homo‐esperetin‐7‐O‐rutinoside.

This chemical discrimination broadly paralleled the biological profiles. Methanol extracts exhibited the strongest antioxidant activity and bactericidal effects against *S. aureus* (ZNSTM) and *B. cereus* (ZNLM), whereas hexane extracts showed weaker antioxidant activity but retained antibacterial effects against Gram‐positive bacteria. An even clearer divergence was observed between the ethyl acetate extracts: ZNLEA showed relatively strong antioxidant activity and was the only extract active against both Gram‐negative bacteria, whereas ZNSTEA displayed weak antioxidant activity despite being bactericidal against *S. aureus*. These contrasting responses indicate that antioxidant and antimicrobial activities are associated with partially distinct chemical profiles and are influenced by both solvent polarity and tissue origin.

VIP analysis identified the metabolites contributing most strongly to extract discrimination. Lignans were prominent, particularly 5′,5′′‐didemethoxypinoresinol (**33**), which showed the highest VIP score, together with pinoresinol (**36**) and sesamin (**39**). Nitrogen‐containing metabolites, including 3‐hydroxydehydroevodiamine (**78**), norchelerythrine (**75**), and 8‐hydroxy‐9‐methoxy‐2,3‐(methylenedioxy)benzophenanthridine (**100**), were also important discriminating features, as were the coumarin‐related metabolites schinilenol (**14**), schinindiol (**16**), and dimethoxysamin (**41**). Flavonoids such as diosmin (**29**) and homo‐hesperetin‐7‐O‐rutinoside (**30**) further contributed to discrimination, together with Wutaipyranol A (**58**), Irenolone (**59**), Musanolone E (**102**), and lipophilic metabolites such as hexadec‐3‐enoic acid (**88**) and 2‐pentadecylfuran (**101**). Thus, differentiation among extracts involves multiple chemical classes rather than a single group of metabolites.

Hierarchical clustering provided complementary information on the distribution of these metabolites (Figure 2). The extracts formed two major groups predominantly according to plant organ, separating stem (ZNSTH, ZNSTEA, and ZNSTM) from leaf extracts (ZNLH, ZNLEA, and ZNLM), while solvent polarity further structured the profiles within each group. Several high‐ranking VIP metabolites were associated with the methanol fractions, including 5′,5′′‐didemethoxypinoresinol (**33**), 3‐hydroxydehydroevodiamine (**78**), 8‐hydroxy‐9‐methoxy‐2,3‐(methylenedioxy)benzophenanthridine (**100**), and dihydroergotamine (**98**). In contrast, 4′‐O‐methylkanzonol W (**23**), norchelerythrine (**75**), cocculine (**81**), schinindiol (**16**), and gibberellin monocarboxylic acid (**69**) characterized a stem‐associated chemical signature, while apigenin (**27**) and isoquercitrin (**26**) contributed to differentiation of the leaf fractions.

**FIGURE 2:**
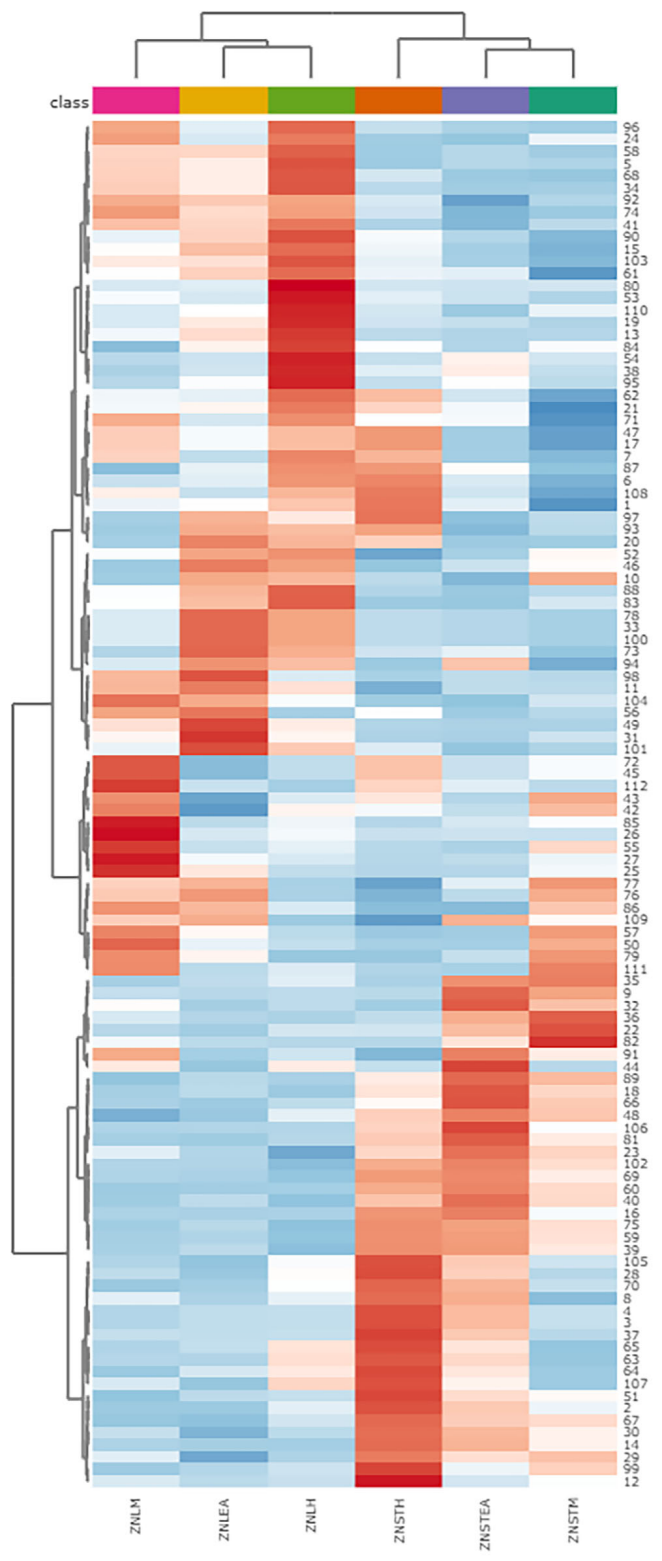
Heatmap based on the compounds identified in *Z. nemorale* extracts.

## 3 Discussion

The present study provides the first integrated metabolomic characterization of *Z. nemorale* leaf and stem extracts in association with their antioxidant and antimicrobial activities, offering new insights into the chemical diversity of this species. Taken together, the biological and metabolomic data indicate that extraction solvent and tissue origin act jointly to determine the chemical and functional profiles of *Z. nemorale*. Solvent polarity defines which portions of the metabolome are preferentially recovered, whereas organ‐specific metabolism determines the composition available for extraction. This interaction is particularly evident from the distinct chemical and biological profiles of leaf and stem fractions obtained with the same solvent.

The association between polar fractions and stronger antioxidant activity may be related to the presence of phenolic‐rich metabolite classes, including lignans and flavonoids. Hydroxylated aromatic structures can stabilize radical intermediates through hydrogen‐atom donation or electron transfer, providing a plausible chemical explanation for the radical‐scavenging activity observed in the methanol fractions [10, 11]. Several putatively annotated lignans, including 5′,5′′‐didemethoxypinoresinol (**33**), pinoresinol (**36**), and sesamin (**39**), were identified among the discriminating metabolites. Flavonoids, including diosmin (**29**) and homo‐hesperetin‐7‐O‐rutinoside (**30**), were also associated with the chemical differentiation of the extracts. However, these associations do not establish their individual contributions to antioxidant activity, which require further experimental validation. Nevertheless, the strong antioxidant activity of ZNLEA demonstrates that antioxidant constituents are not restricted to the most polar fraction and emphasizes the contribution of moderately polar metabolites [11].

The antimicrobial results reveal a broader chemical basis. Unlike antioxidant activity, which was concentrated mainly in polar and moderately polar extracts, antibacterial activity occurred across all three solvent fractions. The retention of activity by hexane extracts suggests that lipophilic constituents can contribute to antibacterial effects even when radical‐scavenging capacity is limited. This interpretation is compatible with the occurrence of discriminating lipophilic metabolites such as hexadec‐3‐enoic acid (**88**) and 2‐pentadecylfuran (**101**), although their individual contributions cannot be established from the present data [12, 13].

Nitrogen‐containing and coumarin‐related metabolites may also contribute to the chemical profiles associated with antibacterial activity. Putatively annotated compounds such as 3‐hydroxydehydroevodiamine (**78**), norchelerythrine (**75**), and 8‐hydroxy‐9‐methoxy‐2,3‐(methylenedioxy)benzophenanthridine (**100**) were among the metabolites contributing to extract discrimination. Coumarin‐related metabolites, including schinilenol (**14**) and schinindiol (**16**), further illustrate the chemical diversity of these fractions. Although these metabolite classes have been associated with antimicrobial properties in previous studies [14, 15, 16], their individual contributions to the activity observed here remain unknown. Consequently, they should be regarded as candidate contributors requiring structural confirmation and experimental validation.

The preferential activity against Gram‐positive bacteria is also compatible with this chemically diverse antimicrobial profile. The absence of an outer membrane makes Gram‐positive cells generally more accessible to compounds acting at the membrane or intracellular level, whereas the additional permeability barrier of Gram‐negative bacteria can restrict entry of many plant‐derived metabolites. The exceptional behavior of ZNLEA is therefore particularly informative. Its ability to inhibit both Gram‐negative species, combined with its relatively strong antioxidant activity, suggests a chemical composition distinct from the other fractions and supports the metabolomic evidence for organ‐specific accumulation of moderately polar constituents. Plant organ emerged as an important source of variation independently of solvent polarity. Hierarchical clustering separated leaf and stem extracts into major groups, while PLS‐DA also distinguished tissues within each solvent system. These patterns indicate tissue‐dependent accumulation of specialized metabolites, which is consistent with the distinct physiological and defensive roles of leaves and stems. However, the data also show that organ effects cannot be considered independently of extraction selectivity: the pronounced differences between ZNLEA and ZNSTEA demonstrate that the same solvent can recover chemically and biologically distinct fractions from different tissues.

The overall metabolite profile is also consistent with the chemically diverse specialized metabolism associated with the genus Zanthoxylum. Coumarins, lignans, flavonoids, phenolic acids, and several nitrogen‐containing metabolites were detected, and representatives of these classes contributed substantially to multivariate discrimination. Their occurrence expands the chemical characterization of *Z. nemorale* and provides a set of candidate markers for subsequent targeted investigation.

Importantly, the correspondence between VIP features, hierarchical clustering, and biological activity should be interpreted as an association rather than direct evidence of causality. VIP scores identify variables that contribute to discrimination among extract groups, but they do not establish that individual metabolites are responsible for antioxidant or antimicrobial effects. Likewise, crude extracts contain complex mixtures in which additive, synergistic, or antagonistic interactions may influence biological responses. The present results therefore provide a rational framework for prioritizing candidate compounds rather than definitive assignment of activity to specific metabolites.

Overall, integration of LC‐HRMS metabolomics with antioxidant and antimicrobial assays reveals two distinct but overlapping bioactivity‐associated chemical trends in *Z. nemorale*. Antioxidant activity is more closely associated with polar to moderately polar, phenolic‐rich profiles, whereas antibacterial activity spans a broader chemical space involving phenolic, nitrogen‐containing, coumarin‐related, and lipophilic constituents. The convergence of biological assays, PLS‐DA, VIP analysis, and hierarchical clustering provides a focused set of candidate metabolites for subsequent structural confirmation, targeted quantification, isolation, and bioactivity‐guided validation, thereby advancing the understanding of the chemical basis of the biological properties of *Z. nemorale*.

Future investigations should prioritize the bioactivity‐guided fractionation of the extracts exhibiting the strongest antioxidant and antimicrobial activities. Active fractions should subsequently undergo chromatographic separation and purification to isolate individual metabolites, followed by structural elucidation using nuclear magnetic resonance (NMR) spectroscopy and high‐resolution mass spectrometry (HRMS). The isolated compounds should then be evaluated individually for antioxidant and antimicrobial activities to establish their contributions to the biological effects observed in the crude extracts. Comparative evaluation of isolated compounds and their combinations could also help identify potential synergistic interactions. This stepwise approach would enable experimental validation of the putative metabolite annotations and provide a clearer understanding of the relationships between chemical composition and biological activity in *Z. nemorale*.

Overall, the integration of LC‐HRMS metabolomics with antioxidant and antimicrobial assays revealed distinct but partially overlapping chemical and biological profiles in *Z. nemorale* extracts. Stronger antioxidant activity was observed in polar and moderately polar fractions, whereas antibacterial activity was distributed across extracts of different polarities. Although multivariate analyses identified several metabolite classes associated with extract discrimination, these associations should not be interpreted as evidence of individual biological activity. The use of crude extracts and putative metabolite annotations limits the assignment of biological effects to specific compounds. Therefore, structural confirmation using complementary analytical techniques, quantitative determination of candidate metabolites, and bioactivity‐guided fractionation and isolation are necessary to establish their individual contributions and potential interactions.

## 4 Conclusion

This study provides an integrated metabolomic and biological characterization of *Z. nemorale*, demonstrating that extraction solvent and plant organ influence its chemical composition and biological activity. LC‐HRMS profiling combined with multivariate analysis revealed distinct metabolomic signatures among the extracts. Methanol fractions exhibited the strongest antioxidant activity and notable antibacterial effects, particularly against Gram‐positive bacteria. Putatively annotated lignans, nitrogen‐containing metabolites, coumarins, flavonoids, and lipophilic constituents contributed to the chemical discrimination among extracts. Although some of these metabolite classes may be associated with the observed biological activities, their individual contributions remain to be established. Structural confirmation, quantitative analysis, and bioactivity‐guided isolation will be essential to validate the proposed associations and identify the compounds responsible for the observed effects. These findings provide a foundation for further investigation of the chemical diversity and biological potential of *Z. nemorale*.

## 5 Experimental Section

### 5.1 Plant Material and Extraction

The study was registered with the Genetic Heritage Management Council (A0F9573). Plant material was collected in October 2019 in the Quilombola community of Três Lagoas, Amargosa, Bahia, Brazil, and identified by Prof. Lidyanne Y. S. Aona. A voucher specimen (HURB 2064) was deposited in the Herbarium of the Recôncavo of Bahia. Leaves and stems were dried at room temperature, separated, and ground using a Willye TE‐680 knife mill. Each plant part was successively macerated with hexane, ethyl acetate, and methanol for 72 h at room temperature. After filtration, the solvents were removed under reduced pressure using a rotary evaporator (Fisatom 550, Brazil), yielding six extracts corresponding to the two plant organs and three extraction solvents.

### 5.2 Determination of Antioxidant Activity

The antioxidant activity of *Z. nemorale* extracts was evaluated using the 2,2‐diphenyl‐1‐picrylhydrazyl (DPPH) radical‐scavenging assay, following the methodology previously described [17], with minor modifications. Extract solutions (500 µL; 0–1000 µg mL^−^^1^) were mixed with 500 µL of DPPH stock solution (120 µmol L^−^^1^ in methanol) and incubated in the dark for 30 min. Absorbance was measured at 515 nm using a microplate reader (VersaMax, USA). A DPPH calibration curve was constructed over the concentration range of 0–60 µmol L^−^^1^. A mixture of DPPH solution and methanol was used as the negative control, and gallic acid was used as the positive control. DPPH consumption was calculated using the calibration curve, and IC_50_ values were determined from the concentration–response curves by linear regression within the appropriate response range. All assays were performed using three independent biological replicates (n = 3), and the results are expressed as mean ± standard deviation (SD).

### 5.3 Antimicrobial Activity

The antimicrobial activity of *Z. nemorale* extracts was evaluated using the broth microdilution method in 96‐well plates, following previously described procedures [18, 19, 20]. The assays were performed against Gram‐positive bacteria [*B. subtilis* (ATCC 6633), *B. cereus* (CCT 0096), *M. luteus* (ATCC 10240), and *S. aureus* (ATCC 6538)], Gram‐negative bacteria [*E. coli* (ATCC 94863) and *P. aeruginosa* (CCT 0090)], and yeasts [*Candida albicans* (ATCC 18804) and *C. glabrata* (CCT 0728)]. Extract concentrations of up to 500 µg mL^−^^1^ were evaluated. Antimicrobial assays were performed using three independent biological replicates (n = 3).

The minimum inhibitory concentration (MIC) was defined as the lowest extract concentration that completely inhibited visible microbial growth. To determine the minimum bactericidal concentration (MBC) and minimum fungicidal concentration (MFC), aliquots from wells showing no visible growth were transferred to agar plates and incubated. MBC and MFC were defined as the lowest concentrations at which no microbial growth was observed after subculturing. Growth following subculture indicated inhibitory activity without a demonstrated bactericidal or fungicidal effect at the tested concentration. Further methodological details are provided in reference [18, 19, 20].

## 6 Metabolite Profiling Analyses

### 6.1 High‐Performance Liquid Chromatography Coupled With High‐Resolution Mass Spectrometry (HPLC‐HRMS)

HPLC‐HRMS analyses were performed using a liquid chromatography system (Prominence, Shimadzu Co., Japan) coupled to a quadrupole time‐of‐flight mass spectrometer (microTOF II, Bruker Daltonics, Germany) [19, 20, 21]. Chromatographic separation was performed on a C18 column (3.0 × 150 mm, 2.6 µm; Kinetex, Phenomenex) at 40°C using water and acetonitrile, both containing 0.1% (v/v) formic acid, at a flow rate of 0.2 mL·min^−^^1^. The gradient consisted of 25% acetonitrile for 2 min, increasing to 80% over 20 min and to 90% over 2 min, followed by re‐equilibration at 25% for 3 min. Mass spectra were acquired in positive and negative ESI modes (3.2 kV; dry gas, 350°C) over an *m/z* range of 50–1000. Dried extracts were dissolved in HPLC‐grade acetonitrile (1 mg·mL^−^^1^), diluted twofold with acetonitrile, and directly injected into the HPLC‐MS system.

### 6.2 Data Processing and Metabolite Identification

HPLC‐HRMS data were processed using XCMS Online to obtain aligned retention times, *m/z* values, and signal intensities. Metabolites were putatively identified using two complementary approaches. First, experimental accurate masses were compared with theoretical values calculated for the main adducts ([M+H]^+^ and [M+Na]^+^ in positive mode and [M−H]^−^ in negative mode), using a mass tolerance of 10 ppm. Theoretical exact masses and mass errors were calculated using online Exact Mass Calculator and University of Warwick tools, respectively. Second, experimental *m/z* values were searched against the Human Metabolome Database (HMDB) according to ionization mode and adduct type ([M+H]^+^, [M+Na]^+^, and [M+NH_3_]^+^ in positive mode; [M−H]^−^ in negative mode), using a mass tolerance of 1 ppm and a Collision Cross Section Tolerance (CCST) of 10. Candidate annotations were evaluated considering mass accuracy, molecular formula, adduct information, and chemical plausibility. Whenever multiple compounds matched the same molecular formula and accurate mass, possible isomeric structures were considered and indicated where applicable. Following the Metabolomics Standards Initiative (MSI), the reported annotations were treated as tentative, with Level 2 assigned. Since MS/MS fragmentation data and authentic reference standards were unavailable, no Level 1 identifications were claimed. Consequently, the proposed compound names represent candidate structures rather than unequivocally identified metabolites. The resulting annotations were used to characterize the chemical diversity of *Z. nemorale* extracts and investigate differences in their metabolomic profiles.

### 6.3 Multivariate Statistical Analysis

Multivariate statistical analyses were performed using the aligned LC‐HRMS dataset obtained from XCMS Online. The experimental dataset comprised six extract groups, corresponding to leaf and stem extracts obtained with hexane, ethyl acetate, and methanol. Each group was represented by 3 independent biological replicates. Partial least squares discriminant analysis (PLS‐DA) was performed to investigate differences in metabolomic profiles according to plant organ and extraction solvent. Model validation was conducted using leave‐one‐out cross‐validation (LOOCV) and permutation testing to assess model robustness and potential overfitting. Model performance was evaluated using the coefficient of determination (R^2^) and cross‐validated predictive ability (Q^2^). [Include permutation testing and classification performance only if these analyses were actually performed.] Variable Importance in Projection (VIP) scores were calculated to identify the metabolomic features contributing most strongly to discrimination among extracts. Hierarchical clustering and heatmap visualization were additionally employed to explore similarities and differences in metabolite distribution across the experimental groups [19, 20, 21].

## Author Contribution

**Victor S. A. de Lira** and **Rodrigo N. Santos** performed the experiments. **Floricéa M. Araújo**, **Lourdes C. Souza‐Neta**, **Leonardo Zambotti‐Villela**, **Pio Colepicolo**, **Lidyanne Y. S. Aona**, and **Paulo R. Ribeiro** provided support for the experiments and data acquisition. **Paulo R. Ribeiro** and **Floricéa M. Araújo** supervised the work. **Paulo R. Ribeiro** and **Victor S. A. de Lira** wrote the manuscript. All authors have read and approved the final version of the manuscript.

## Conflicts of Interest

The authors declare no conflicts of interest.

## Use of Generative AI and AI‐Assisted Technologies in the Writing Process

Generative AI (chatgpt) was used exclusively for grammar correction, language polishing, and minor improvements in word choice. It was not used to generate substantial scientific content, restructure arguments.

## Supporting information



Supporting information for this article is available on the WWW under https://doi.org/10.1002/cbdv.71883.
**Supporting File 1**: cbdv71883‐sup‐0001‐SuppMat.docx

## Data Availability

The data that support the findings of this study are available from the corresponding author upon reasonable request.
