# Supplementary material for: Metabolite Profile and Biological Activities of Zanthoxylum nemorale: A Metabolomic and Multivariate Statistical Analysis Study
Source: Chem Biodivers. 2026 Oct 27;23(10):e71883. doi: 10.1002/cbdv.71883 (PMC13711469; doi:10.1002/cbdv.71883)
Supplement: Supplementary file 1 [file CBDV-23-e71883-s001.docx]

**Supplementary Figure 1**. Chemical structures of compounds **1-32** identified in *Z. nemorale* extracts.

**Supplementary Figure 2**. Chemical structures of compounds **32-58** identified in *Z. nemorale* extracts.

**Supplementary Figure 3**. Chemical structures of compounds **59-85** identified in *Z. nemorale* extracts.

**Supplementary Figure 4**. Chemical structures of compounds **86-112** identified in *Z. nemorale* extracts
